# Significantly Enhanced Balance of Dielectric Properties of Polyvinylidene Difluoride Three-Phase Composites by Silver Deposited on K_2_Ni_0.93_Ti_7.07_O_16_ Hollandite Nanoparticles

**DOI:** 10.3390/polym16020223

**Published:** 2024-01-12

**Authors:** Alexey Tsyganov, Maria Vikulova, Ilya Zotov, Denis Artyukhov, Igor Burmistrov, Alexander Gorokhovsky, Nikolay Gorshkov

**Affiliations:** 1 Department of Chemistry and Technology of Materials, Yuri Gagarin State Technical University of Saratov, 77 Polytecnicheskaya Street, 410054 Saratov, Russia; 2 Department of Power and Electrical Engineering, Yuri Gagarin State Technical University of Saratov, 77 Polytecnicheskaya Street, 410054 Saratov, Russia; 3 Engineering Center, Plekhanov Russian University of Economics, 36 Stremyanny Lane, 117997 Moscow, Russia

**Keywords:** ceramics, hollandite, dielectric properties, polyvinylidene difluoride, high-k

## Abstract

Three-phase polymer composites are promising materials for creating electronic device components. The qualitative and quantitative composition of such composites has a significant effect on their functional, in particular dielectric properties. In this study, ceramic filler K_2_Ni_0.93_Ti_7.07_O_16_ (KNTO) with Ag coating as conductive additive (0.5, 1.0, 2.5 wt.%) was introduced into the polyvinylidene difluoride (PVDF) polymer matrix in amounts of 7.5, 15, 22.5, and 30 vol.%. to optimize the dielectric constant and dielectric loss tangent. The filler was characterized by X-ray phase analysis, Fourier-transform infrared spectroscopy and Scanning electron microscopy methods. The dielectric constant, dielectric loss tangent, and conductivity of three-phase composites KNTO@Ag-PVDF were studied in comparison with two-phase composites KNTO-PVDF in the frequency range from 10^2^ Hz to 10^6^ Hz. The dielectric constant values of composites containing 7.5, 15, 22.5, and 30 vol.% filler were 12, 13, 17.4, 19.2 for pure KNTO and 13, 19, 25, 31 for KNTO@Ag filler (2.5 wt.%) at frequency 10 kHz. The dielectric loss tangent ranged from 0.111 to 0.340 at a filler content of 7.5 to 30 vol.%. A significantly enhanced balance of dielectric properties of PVDF-based composites was found with K_2_Ni_0.93_Ti_7.07_O_16_ as ceramic filler for 1 wt.% of silver. Composites KNTO@Ag(1 wt.%)-PVDF can be applied as dielectrics for passive elements of flexible electronics.

## 1. Introduction

In the conditions of modern development of the electrical industry, dielectric materials are in extreme demand for the manufacture of various electronic components [1,2,3,4]. Among them, polymers have attracted much attention due to the ease of manufacturing electronic components of various shapes. In addition, polymers have low dielectric losses, high breakdown strength, and can be used in flexible electronics [5,6,7,8,9,10,11]. However, polymers have a very low dielectric constant (*ε*′ = 2–12), which greatly limits their application. The most advanced polymer for dielectric applications is poly (vinylidene fluoride) (PVDF) due to its high dielectric constant (*ε*′ ≈ 10) [12]. In addition to its dielectric constant, high for polymers, PVDF is characterized by high breakdown voltage and low dielectric loss tangent. Moreover, PVDF is low cost and easy to manufacture, making it an excellent candidate for the additive manufacturing of electronic components by extrusion method [13,14]. Therefore, a large number of studies are aimed at obtaining composites based on a PVDF matrix with an improved set of dielectric characteristics [15,16,17,18,19,20]. Dielectric characteristics primarily include dielectric constant as a quantity characterizing polarization under the influence of an electric field and dielectric loss tangent, which quantitatively characterizes the dissipation of electrical energy due to physical processes of various natures. The PVDF polymer matrix is most in demand for energy storage devices and capacitive sensors, where dielectric constant is a quantitative measure of stored energy and sensor sensitivity, respectively. In addition, it is extremely important to ensure a low dielectric loss tangent to increase efficiency and avoid excessive heating of the manufactured devices. Thus, the developed polymer composites must have a balance of dielectric constant and dielectric loss tangent that is optimal for their practical application. For this purpose, various fillers and approaches of their modification have been investigated.

Ceramic or conductive fillers are usually introduced into polymer matrices to improve their dielectric properties. Materials with high dielectric constant, for example (Ba, Sr)TiO_3_ [21,22,23], CaCu_3_Ti_4_O_12_ [24,25,26,27], TiO_2_ [28,29,30], LaFeO_3_ [31,32], are commonly used as ceramic fillers in polymer composites [33,34]. A large volume fraction of ceramic filler (usually *f* > 50 vol.%) is needed to significantly increase the dielectric constant of composites, which inevitably leads to a loss of flexibility and difficulties in molding. An increase in the filler loading in composites is always accompanied by an increase in dielectric losses in addition to an increase in the dielectric constant. An important factor determining the properties of composites is the interfacial interaction of the filler with the polymer matrix. Therefore, it is extremely important to ensure good interfacial adhesion between the ceramic particles and the polymer matrix [35]. Considering the potential for low filler loading, conductive fillers such as carbon nanomaterials [36,37], metal nanoparticles [38,39,40,41], and MXenes [42,43,44] are of greater interest. In this case, a significant increase in the dielectric constant occurs near the percolation threshold; however, the conducting particles inevitably form conducting paths, which leads to an increase in the conductivity of the composites and high dielectric losses. Despite the disadvantages of ceramic and conductive fillers for polymer matrices, their simultaneous introduction into composites makes it possible to improve dielectric properties [45,46,47,48]. In particular, one of the effective approaches is the deposition of conductive particles on the surface of ceramic particles [49,50]. Silver nanoparticles are of the greatest interest as conductive particles deposited on the surface of ceramics [51,52,53,54]. This approach makes it possible to improve the polarization of the space charge and to achieve a higher performance of the dielectric properties of composites [55].

In recent years, potassium titanates K_x_Ti_8-y_Me_y_O_16_ with the hollandite structure have been of particular interest due to the possibility of their modification and a wide range of applications, including electrode materials for energy storage devices, gas sensors, catalysts, etc. [56,57,58,59,60]. In addition, recent publications report that hollandite-like solid solution ceramics can exhibit high dielectric constants (*ε*′ = 10^3^–10^4^) [61,62], which also makes them attractive materials as ceramic fillers for high dielectric polymer composites. Previously, hollandite-like solid solutions K_x_Ti_8-y_Me_y_O_16_ (Me = Cu, Ni, Fe) were studied as fillers for PMMA, Epoxy, PVDF, and PTFE matrices [63,64,65,66]; however, these composites demonstrated an increase in dielectric constant and strong increase in dielectric losses. An effective way to inhibit dielectric losses in the case of PTFE and PVDF polymer matrices was to use a three-phase strategy in the preparation of composites. The third phase was carbon nanotubes and carbon black distributed on the surface of hollandite-like particles. Based on the fact that three-phase composites with the participation of various ceramic particles and silver nanoparticles demonstrate balanced values of dielectric constant and dielectric loss tangent, it can be assumed that modification of the surface of potassium titanates with the hollandite structure with silver nanoparticles can make it possible to achieve improved dielectric properties.

The aim of this research is to study the dielectric properties of composites based on a PVDF matrix with the addition of a K_x_Ti_8-y_Ni_y_O_16_ (KNTO) ceramic filler, the surface of which is modified with silver nanoparticles at various concentrations.

## 2. Materials and Methods

### 2.1. KNTO Powder Synthesis

**Figure 1:**
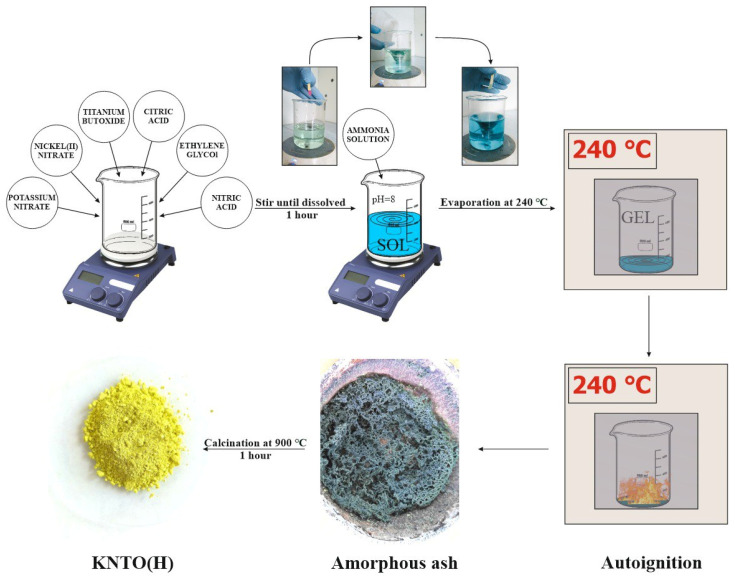
Scheme of KNTO powder synthesis.

Nanosized KNTO powder was obtained by the modified Pechini sol-gel method as shown in Figure 1 using the following materials: titanium isopropoxide (C_16_H_36_O_4_Ti, 97%, Acros Organics, Geel, Belgium), potassium nitrate (KNO_3_, purity of 98%, Buyskiy himicheskiy zavod, Buy, Russia), nickel nitrate (Ni(NO_3_)_2_·6H_2_O, purity of 98%, Buyskiy himicheskiy zavod, Buy, Russia), citric acid (C_6_H_8_O_7_·2H_2_O purity of 99.5%, Aricon, Moscow, Russia), nitric acid (HNO_3_, 65% aqueous solution, Buyskiy himicheskiy zavod, Buy, Russia), ammonia (NH_4_OH, 25 wt.%, Reahim, Moscow, Russia), ethylene glycol (C_2_H_6_O_2_, purity of 98.5%, Aricon, Moscow, Russia). This method is based on the polymerization of metal citrate using ethylene glycol. Citric acid is used as a cation chelating agent in aqueous solution, and ethylene glycol leads to the formation of an organic ester. Polymerization stimulated by heating the mixture leads to the formation of a homogeneous resin, in which metal ions are evenly distributed throughout the organic matrix. The synthesis was carried out according to the procedure in research [62].

To obtain KNTO powder, an aqueous solution containing citric acid, ethylene glycol, potassium and nickel nitrates was added to titanium isopropoxide with stirring, then nitric acid was added until all components were completely dissolved in the reactor. The molar ratios of the cations of the corresponding metals varied in accordance with the chemical formula of potassium titanate with hollandite structure K_y_Ni_x_Ti_8−x_O_16_ in the ranges of 0.5 ≤ *x* ≤ 1, 1 ≤ *y* ≤ 2. However, the single-phase powder with the holladite structure used later was obtained only at the ratio of the corresponding chemical formula K_2_Ni_0.93_Ti_7.07_O_16_; in other cases, TiO_2_ and K_2_Ti_6_O_13_ impurity phases were observed. Citric acid and ethylene glycol were added in respective proportions of 5 and 1.5 mol for each mol of titanium cation. Then, an ammonia solution was added to the obtained mixture until pH = 8 was established, as a result of which the mixture acquired a dark green color. The resulting mixture was heated at 250 °C to form a viscous polymer product and then an amorphous ash resulting from autoignition. The amorphous ash was calcined at 900 °C for 1 h in an air atmosphere to obtain a yellow KNTO powder. For further modification and production of polymer composites, KNTO powder was ground in a Fritsch Pulverisette 0 vibrating micromill to break up the agglomerated particles.

### 2.2. KNTO@Ag Powder Preparation

The surface of KNTO particles was modified with silver nanoparticles using the following technique. Weighted portions of silver nitrate (AgNO_3_, 99.9%, LenReaktiv, Saint-Petersburg, Russia) corresponding to 0.5, 1, and 2.5 wt.% Ag in relation to the mass of KNTO ceramic powder were dissolved in distilled water. Then, an ammonia solution (NH_4_OH, 25 wt.%, Reahim, Moscow, Russia) was added to the resulting solutions until the brown precipitate was completely dissolved. Aqueous dispersions of KNTO powders with a concentration of 5 wt.% were added to the resulting solutions. An aqueous dispersion of KNTO was obtained by ultrasonic treatment of the powder in deionized water for 2 h using an ultrasonic bath with a power of 100 W. Further, equivalent amounts of a 40% formalin solution were added dropwise to the dispersions with constant stirring to completely reduce silver ions to nanoparticles of metallic silver. Upon reduction of silver ions, the green KNTO dispersion acquired a dark color. The resulting KNTO@Ag powders were washed with distilled water and then dried in an electric oven at 80 °C for 2 h.

### 2.3. Preparing Composites Method

To prepare three-phase KNTO@Ag-PVDF polymer composites, a 2 wt.% solution of poly (vinylidene fluoride) (PVDF, Sigma Aldrich, Mw~530,000, St. Louis, MO, USA) was first prepared in dimethylsulfoxide ((CH_3_)_2_SO, 99.5%, VitaHim, Dzerzhinsk, Russia), to which the corresponding KNTO@Ag powders were added in amounts of 7.5, 15, 22.5, and 30 vol.%. The theoretical density value of 3.878 g/cm^3^, calculated using the Rietveld method, was used to calculate the mass weights of KNTO powder. For homogenization, the resulting dispersions were subjected to mechanical stirring on a magnetic stirrer for 24 h, followed by ultrasonic treatment for 1 h. The prepared mixtures were poured into deionized water and dried at 100 °C for 10 h to remove the solvent. From the obtained composites, disks 12 mm in diameter and 1 mm in thickness were formed using uniaxial hot pressing at a temperature of 200 °C and a pressure of 20 MPa. Conductive silver paste was applied to the obtained composite products as electrodes.

### 2.4. Characteristic Methods

The ARL X’TRA device (Thermo Scientific, Ecublens, Switzerland) with Cu Kα radiation (*λ* = 0.15412 nm) was used in the diffraction studies of KNTO and KNTO@Ag. The lattice parameters were refined using the Rietveld method. An FT-801 FTIR spectrometer (Simex, Novosibirsk, Russia) was used for Fourier transform infrared spectroscopy (FTIR). The morphology of KNTO@Ag particles was investigated using an ASPEX Explorer scanning electron microscope (ASPEX, Framingham, MA, USA). The fractional composition of the powder was studied using laser particle size analyzer ANALYSETTE 22 MicroTec plus (FRITSCH, Idar-Oberstein, Germany). A Novocontrol Alpha AN Impedance Analyzer (Novocontrol Technologies GmbH & Co. KG, Montabaur, Germany) was used to determine the dielectric characteristics of the nanocomposites. The values of dielectric constant (*ε*, *ε*′ and *ε*″), dielectric loss (*tanδ*) and conductivity (*σ*, *σ*′ and *σ*″) were calculated from the obtained experimental data of real (*Z*′) and imaginary (*Z*″) parts of impedance. For reproducibility of the result, the measurements were carried out on three samples of each composition. The frequency range of the measurements was from 10^2^ to 10^6^ Hz at a voltage amplitude of 100 mV.

## 3. Results and Discussion

**Figure 2:**
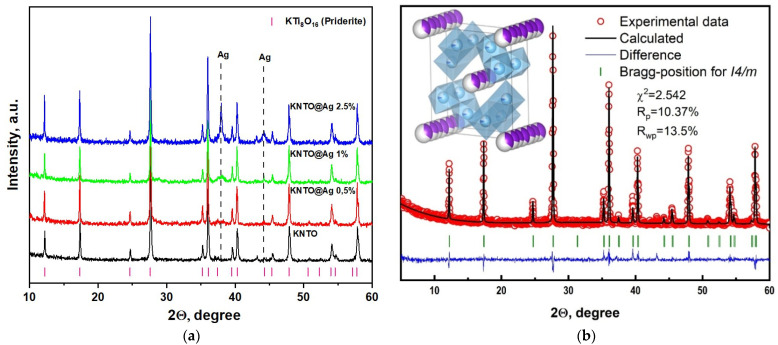
(**a**) Combined X-ray diffraction patterns and (**b**) Rietveld refinement of KNTO: red circles are experimental data, black line is calculated line, blue line is difference, green vertical lines are Bragg positions.

Successful synthesis and modification of ceramic filler for PVDF matrix are confirmed using the X-ray phase analysis method. The diffraction pattern, as well as the Rietveld structural refinements, performed using the General Structure Analysis System software (GSAS-II) for the space group l4/m (ICDD 77-0990) are shown in Figure 2. As can be seen, narrow diffraction reflections of high intensity, attributed to hollandite-like potassium titanate, can be observed in the diffraction pattern. In addition, no reflections corresponding to secondary phases are observed, suggesting that the product is a single-phase KNTO powder. The plot of the final Rietveld refinement shows a good agreement between the experimental and calculated intensities in the tetragonal system with space group I4/m. The calculated cell parameters are *a* = 10.1371 Å and *c* = 2.9629 Å. Theoretical density is 3.878 g/cm^3^.

Figure 2b shows X-ray diffraction patterns of KNTO@Ag powders at various silver contents. As can be seen, when the KNTO powder is treated with silver by deposition reaction, X-ray diffraction patterns show the appearance of reflection peaks, confirming the presence of the silver metal phase. In addition, reflection peaks for the silver phase show a tendency to increase in intensity with increasing concentration, which confirms the successful deposition of silver particles on the KNTO surface.

**Figure 3:**
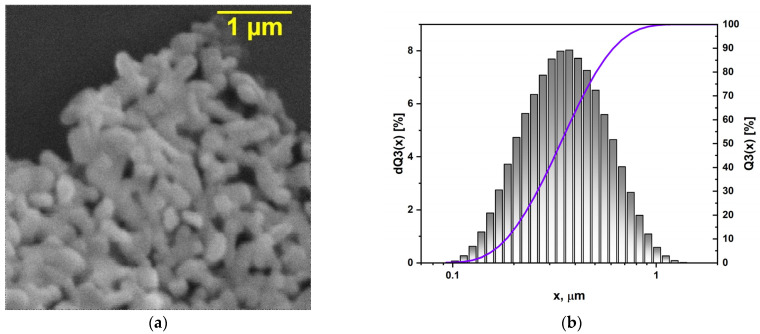
(**a**) The SEM image and (**b**) particle size distribution plot of the KNTO@Ag powder.

To select and adapt the approach of introducing a filler into a polymer matrix when creating composites, it is important to know the morphology and size of its particles. Figure 3 shows the morphology and particle size distribution plot of the KNTO@Ag powder treated with silver nanoparticles at a concentration of 2.5 wt.%. As can be seen, the particle size distribution is a unimodal spectrum with an average particle size *d*_50_ = 350 nm. The SEM image of the powder shows a tetragonal microstructure characteristic of the hollandite structure. It should be noted that SEM images fail to detect the presence of metal additive, which is due to the fact that silver is located on the surface of KNTO in the form of nanoparticles of 10–20 nm in size. The small particle size and relatively narrow particle size distribution of KNTO@Ag makes it possible to predict a uniform distribution of filler particles in the bulk of the polymer matrix.

**Figure 4:**
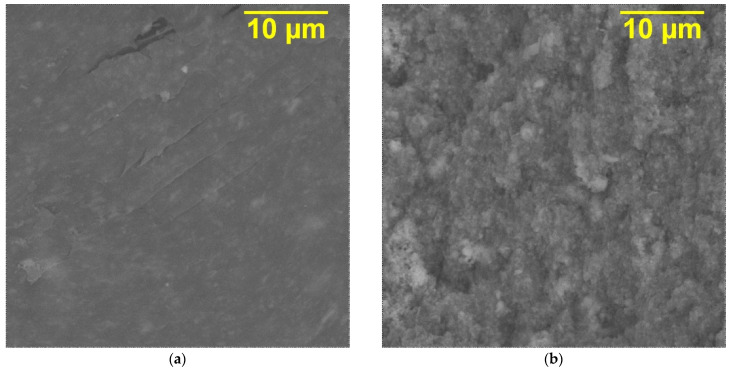
Secondary electron SEM images of PVDF-based composites cross-section with (**a**) 15 vol.% KNTO@Ag (1 wt.%) and (**b**) 30 vol.% KNTO@Ag (2.5 wt.%).

The functional, in particular dielectric, properties of polymer composites depend on the distribution of filler in the matrix structure. In the production of composites of various compositions, a significant amount of attention is paid to the choice of method and conditions for introducing the filler for its most uniform distribution in the polymer. Electron micrographs (Figure 4) show a uniform distribution of filler in the polymer matrix. The more filler, the less particles of the cross-section are covered with polymer. The lighter areas in the secondary electron SEM images most likely belong to the KNTO filler with silver nanoparticles. A pattern can be observed: the higher the concentration of the ceramic filler and conductive additive, the more light inclusions in the microphotograph.

**Figure 5:**
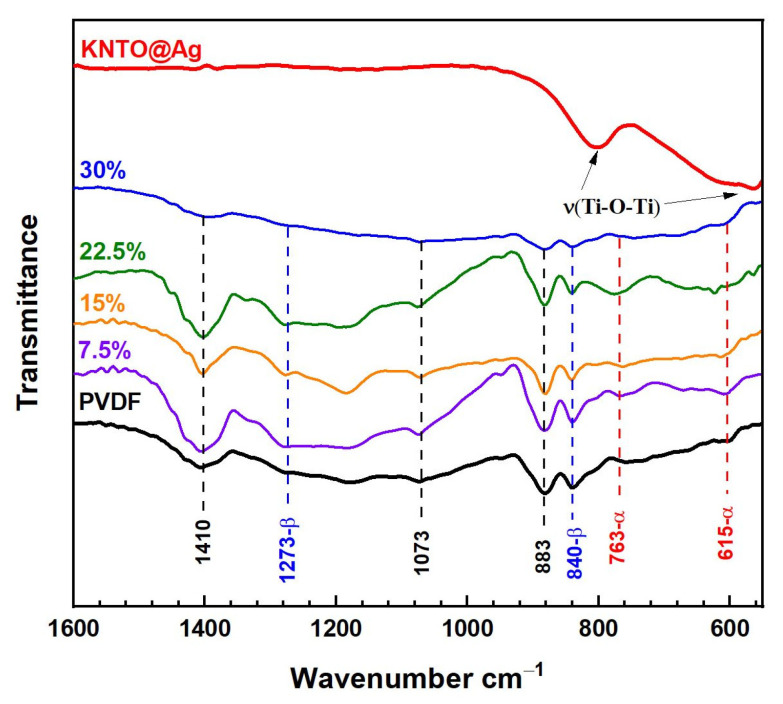
FTIR transmittance spectra of pure PVDF, KNTO@Ag and KNTO@Ag-PVDF composites.

The resulting three-phase composites are qualitatively studied by FTIR spectroscopy in comparison with the individual components of the composite. The absorption bands in the FTIR spectra can be used to identify the presence of the filler and the matrix in polymer composites and to semi-quantitatively estimate the change in the KNTO@Ag volume fraction. Figure 5 shows the FTIR transmittance spectra of pure PVDF, KNTO@Ag and KNTO@Ag-PVDF composites with varying filler fraction. The non-polar α-phase corresponds to the characteristic absorption bands at 615 and 763 cm^−1^. The presence of a ferroelectric β-phase is confirmed by peaks at 840 and 1273 cm^−1^. At the same time, the bands at 883, 1073 and 1410 cm^−1^ have similar characteristics for α-, β- and γ-phases of PVDF [67,68]. Stretching vibrations of the Ti-O-Ti bonds of the filler are characterized by absorption bands at 570 and 805 cm^−1^.

**Figure 6:**
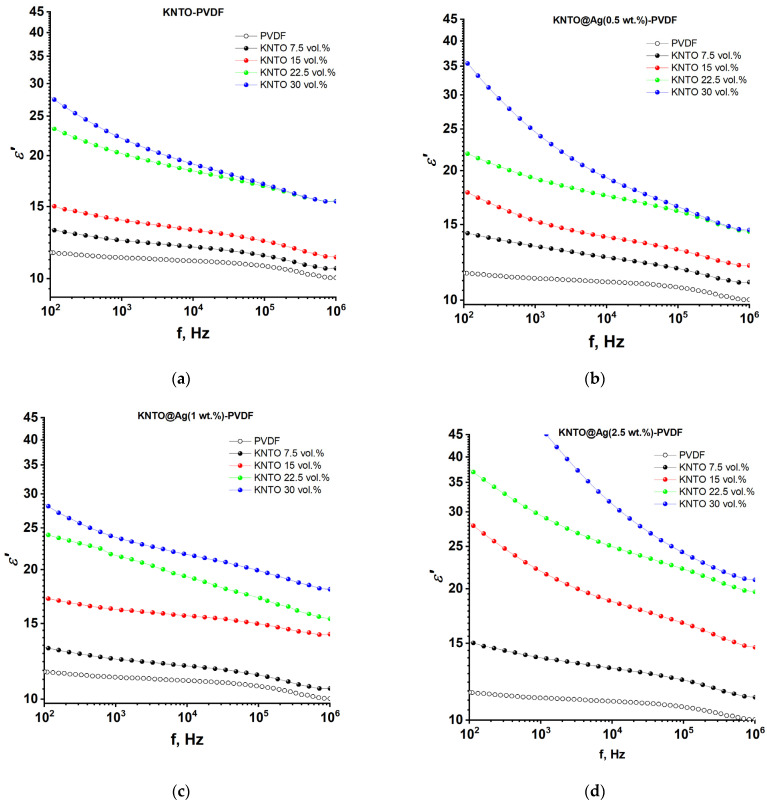
The frequency dependences of the dielectric constant of (**a**) KNTO-PVDF, (**b**) KNTO@Ag(0.5 wt.%)-PVDF, (**c**) KNTO@Ag(1 wt.%)-PVDF, and (**d**) KNTO@Ag(2.5 wt.%)-PVDF composites.

In order to reveal the effect of silver coating on the surface of KNTO particles on the dielectric properties of polymer composites, two compositions of composites were prepared, namely KNTO-PVDF and KNTO@Ag-PVDF. The frequency dependences of the dielectric constant of the KNTO-PVDF and KNTO@Ag-PVDF composites for various volume fractions of the KNTO filler and various mass concentrations of Ag nanoparticles at a frequency range from 10^2^ to 10^6^ Hz are shown in Figure 6. For a pure PVDF polymer matrix, the dielectric constant is *ε*′ = 12. The filler introduction, as expected, promotes an increase in the dielectric constant of the composites as its concentration increases, since the KNTO powder is a ceramic material, the dielectric constant of which is much higher than that of the PVDF matrix. The dielectric constant of the KNTO-PVDF composite is *ε*′ = 12.6 at a volume fraction of KNTO *f* = 7.5 vol.%, and as the concentration increases to *f* = 30 vol.%, the dielectric constant increases to *ε*′ = 19.2 at a frequency of 10 kHz. The increase in dielectric constant in this case is primarily due to the accumulation of electric charge carriers at the KNTO-PVDF interface, leading to a strong Maxwell–Wagner–Sillar polarization effect [35]. The dielectric constant of the three-phase KNTO@Ag-PVDF composites also shows a tendency to increase with an increase in the Ag concentration in filler composition. This trend is more noticeable at filler volume fractions *f* = 15 vol.% and higher. The corresponding dielectric constant values of composites containing 7.5, 15, 22.5, and 30 vol.% filler are 12, 13, 17.4, and 19.2 for pure KNTO and 13, 19, 25, and 31 for the KNTO@Ag filler (2.5 wt.%) at a frequency of 10 kHz. The improved dielectric response upon treatment of the ceramic filler with silver nanoparticles is primarily due to the additional contribution of the microcapacitor effect to the dielectric constant [51]. In this case, the PVDF polymer matrix and KNTO ceramic particles can act as a dielectric, while silver nanoparticles act as an electrode. The capacitance of such microcapacitors depends on the thickness of the dielectric layer between the conductive particles. Depending on the total content of fillers and on the amount of Ag nanoparticles in their composition, one can observe a different nature of the frequency dependence of the dielectric constant. The dielectric constant of a pure PVDF matrix has a weak frequency dependence in the range of 10^2^–10^5^ Hz and shows a sharp decrease with increasing frequency up to 10^6^ Hz, which is mainly a relaxation stage associated with a decrease in polarization due to the transition from low-frequency to high-frequency dipole polarization. In this case, the decrease in the dielectric constant is due to the fact that the dipoles do not have time to quickly change their orientation at high frequencies [53]. The addition of a ceramic filler at concentrations of 7.5 and 15 vol.% does not lead to a strong frequency dependence of the dielectric constant; however, a further increase in the filler concentration leads to a significant increase in the dielectric constant at low frequencies, which is usually associated with a higher dielectric constant of the filler and a high contribution of slow electrical dipoles induced by interfacial polarization. It should be noted that when the surface of KNTO particles is treated with silver nanoparticles (1 wt.%), a weaker frequency dependence of the dielectric constant can be observed, together with its increase, relative to composites with pure KNTO. This may be due to the predominance of the effect of microcapacitor contribution to the dielectric constant of composites at high frequencies. An increase in the silver concentration on the surface of ceramic particles, along with a high dielectric constant, leads to the appearance of a strong frequency dependence of the dielectric constant even at low filler concentrations. This is due to the occurrence of a strong Maxwell–Wagner–Sillars interfacial polarization, since a large number of conductive Ag particles interacting with each other and separated by a dielectric layer of PVDF and KNTO leads to the appearance of a large number of interfaces on which an electric charge accumulates, leading to a strong polarization effect.

**Figure 7:**
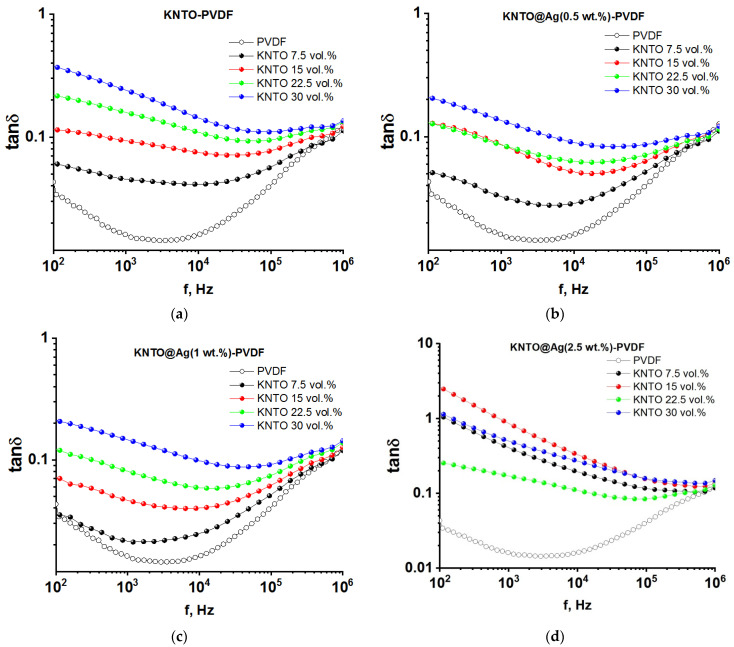
The frequency dependences of the dielectric loss tangent of (**a**) KNTO-PVDF, (**b**) KNTO@Ag(0.5 wt.%)-PVDF, (**c**) KNTO@Ag(1 wt.%)-PVDF, and (**d**) KNTO@Ag(2.5 wt.%)-PVDF composites.

The frequency dependences of the dielectric loss tangent for KNTO-PVDF and KNTO@Ag-PVDF composites are presented in Figure 7. As can be seen, all studied samples show a tendency for the dielectric loss tangent to decrease with increasing frequency from 0.1 kHz to 10 kHz, and then a sharp increase, which is a feature of the relaxation of the PVDF polymer matrix [69,70]. In the case of PVDF, the occurrence of dielectric losses is associated with leakage current losses, which dominate at frequencies below 1 kHz and losses associated with dielectric relaxation at frequencies above 100 kHz. The latter is due to the fact that with increasing frequency, dipoles are delayed in reorientation in an external electric field [71,72,73,74]. It should be noted that the frequency dependence of the dielectric loss tangent can change its behavior depending on the nature and concentration of the filler. An increase in the filler concentration leads to an increase in Maxwell–Wagner–Sillar polarization and an increase in the interfacial leakage current. The dielectric loss of a pure PVDF matrix is 0.015 at a frequency of 10 kHz. With the introduction of a KNTO filler in volume fractions from 7.5 to 30 vol.%, a sharp increase in dielectric losses from 0.041 to 0.147 at a frequency of 10 kHz is observed, which is explained by an increased leakage current from PVDF/KNTO phase boundaries. Modification of the KNTO particle surface with silver nanoparticles makes it possible to reduce the dielectric loss tangent in three-phase KNTO@Ag-PVDF composites relative to two-phase KNTO-PVDF composites at identical filler volume contents. A decrease in dielectric losses of composites is observed for silver concentrations from 0.5 to 1 wt.%. For the KNTO@Ag(1wt.%)-PVDF composite, the dielectric losses range from 0.024 to 0.100 at a filler volume content of 7.5 to 30 vol.% and frequency of 10 kHz. This behavior can be explained by the improvement in the insulating ability of KNTO@Ag-PVDF composites and the high interface compatibility. However, a further increase in the silver concentration to 2.5 wt.% leads to a strong increase in the dielectric loss to values from 0.111 to 0.340 at a filler content of 7.5 to 30 vol.% and at a frequency of 10 kHz. This is due to the fact that an increase in the silver concentration leads to a strong tunneling effect, according to which particles isolated from each other can electrically interact when approaching a critical distance. The occurrence of the tunneling effect, in turn, leads to an increase in conductivity, which continues to increase due to the approach of conducting particles with an increase in the filler loading in the polymer matrix.

**Figure 8:**
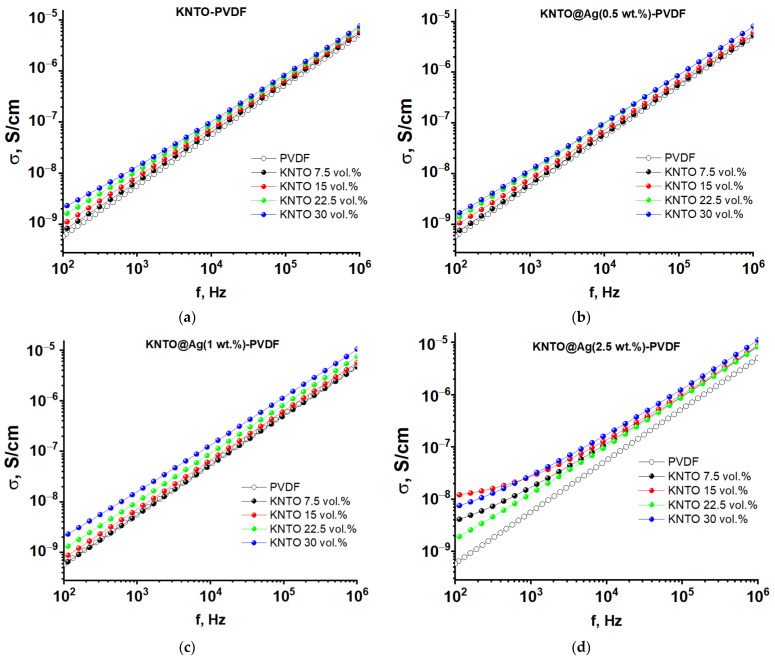
The frequency dependences of the conductivity of (**a**) KNTO-PVDF, (**b**) KNTO@Ag(0.5 wt.%)-PVDF, (**c**) KNTO@Ag(1 wt.%)-PVDF, and (**d**) KNTO@Ag(2.5 wt.%)-PVDF composites.

The dependences of the conductivities of KNTO-PVDF and KNTO@Ag-PVDF composites are shown in Figure 8. In all cases, with an increase in filler loading, an increase in conductivity is observed due to an increasing number of electrons and structural defects with an increase in the volume of the KNTO ceramic filler in the PVDF matrix. The modification of KNTO particles with silver has a significant effect only at a concentration of 2.5 wt.%. In this case, a non-linear relationship is observed with an inflection point at ~100 Hz. Such a sharp increase in conductivity in the low-frequency region is associated with a significant increase in the amount of electrically conductive filler in the bulk of the composites, which leads to the formation of electrically conductive paths that facilitate electron transfer.

**Figure 9:**
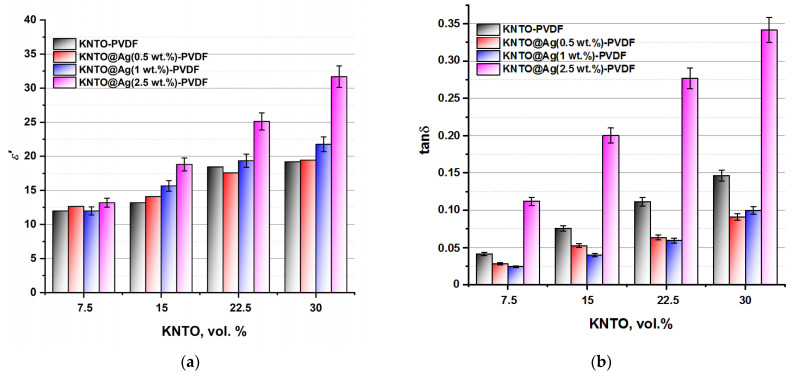
The dependences of (**a**) the dielectric constant and (**b**) dielectric loss tangent of PVDF-based composites with different composition at 1 kHz.

A visual graphical representation of the combined effect of the concentration of ceramic filler KNTO and the content of silver on its surface on the main dielectric characteristics is presented in the form of diagrams in Figure 9. It can be noted that at a higher content of KNTO (15–30 vol.%) in the composition of composites, a more significant effect of the conductive silver coating on the dielectric constant is observed. At the same time, 1 and 2.5 wt.% addition of silver leads to a significant increase in the ε’ value compared to composites with no additive and its minimum amount. In the case of dielectric losses, the following trend can be observed: a decrease in the dielectric loss tangent with the addition of silver of 0.5 and 1 wt.% and its sharp increase in the case of maximum loading (2.5 wt.%). This behavior of polymer matrix composites with fillers of various natures can be explained by the effect of amplifying polarization when an electric field is applied, which leads to an increase in the dielectric constant. In addition, the observed decrease in the dielectric loss tangent is associated with the suppression of free electrons in the area of the interface between the KNTO@Ag filler and the PVDF matrix, which leads to an improvement in the insulating properties of the composites [47]. In general, it can be noted that a simultaneous increase in the dielectric constant and a decrease in dielectric losses is achieved by improving the compatibility of interfaces between the KNTO@Ag filler and the PVDF matrix.

**Figure 10:**
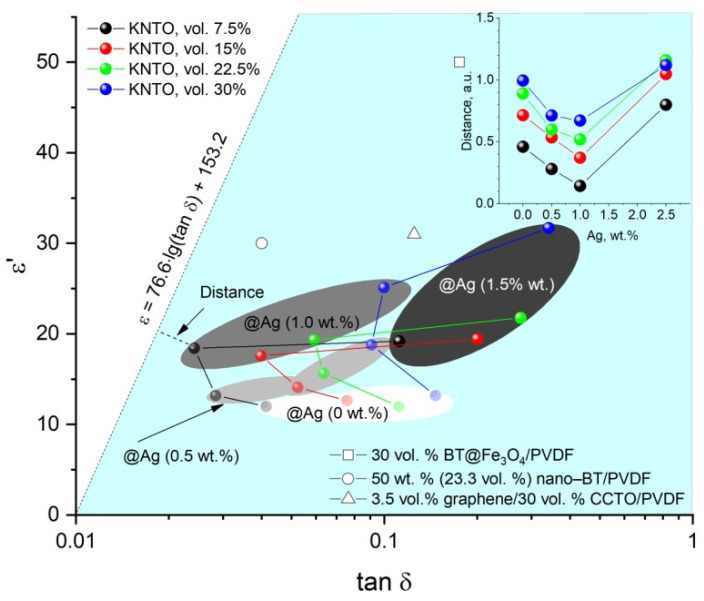
Optimization of the dielectric constant and dielectric loss tangent for three-phase composites based on PVDF at 1 kHz (the blue background is an area of low balance for ε′ and *tanδ*). The color intensity of the balls on the main diagram is determined by the concentration of the silver additive: the more intense the color, the higher the silver content (marked with areas). Data from literary sources: square—[75], circle—[76], triangle—[77]. Inset is the distance between the values of the experimental points and the straight line dividing the diagram.

A common approach for finding the optimal concentrations of ceramic and conductive fillers in a polymer matrix for the improved combination of high dielectric constant and low dielectric loss is the straight line described by the equation in Figure 10. The minimum distance to this straight line is considered to be the optimal combination of the dielectric properties of three-phase composites due to the synergistic effect of KNTO and Ag. It can be noted that the desired effect is provided by a 1 wt.% addition of silver to the ceramic filler. Moreover, the positive effect of this amount of silver can be traced for all volume fractions of KNTO.

Optimization of the values of the dielectric constant and the dielectric loss tangent is the main task for creating composite dielectrics with a polymer matrix. Previously, we used the *ε*′–*tanδ* diagram [66] to analyze the balance of dielectric properties. The diagram is divided by a straight line described by equation *ε* = 76.6 *log*(*tanδ*) + 153.2 from a recently published paper [78]. So, the area up to this line (unshaded) has dielectric values that are satisfactory for use in electronic devices.

The results obtained (Figure 10) are in the shaded region, which shows a suboptimal balance of dielectric properties. However, the distance between the values of the experimental points and the straight line dividing the diagram (inset in Figure 10) demonstrates the minima for composites for all values of the ceramic filler volume fractions at a silver coating concentration of 1 wt.%. Since ceramic filler material has ionic conductivity, high values of the dielectric loss tangent are caused by polaron conductivity. It can be assumed that the applied coating of the conducting silver layer localizes the electrons involved in this process, which leads to optimization of the balance of dielectric properties to a concentration of 1 wt.%. A high silver content of 2.5 wt.% leads to an increase in electronic conductivity over the deposited layer, which greatly increases dielectric losses and a decrease in the balance of dielectric properties, demonstrated by increasing the distance to the region of optimal values (inset in Figure 10). The resulting composites can be used as dielectrics for passive elements of flexible electronics.

## 4. Conclusions

In this work, a ceramic material in the form of potassium titanate K_2_Ni_0.93_Ti_7.07_O_16_ was synthesized by the sol-gel method and modified with a silver coating for use as filler for a PVDF polymer matrix. The resulting ceramic is a single-phase material with a unimodal particle size distribution with *d*_50_ = 350 nm. The presence of a silver additive is confirmed by the corresponding reflections on X-ray diffraction patterns. The filler was introduced into the PVDF polymer in amounts of 7.5, 15, 22.5, and 30 vol.% without and with the addition of silver in amounts 0.5, 1.0, and 2.5 wt.%. A change in the quantitative ratio of the filler/matrix was noted both in the X-ray diffraction patterns and in the FTIR spectra by the intensity of the corresponding reflections and absorption bands. The frequency dependences of the dielectric constant of the obtained polymer composites were of a similar nature and represented curves with a smooth increase in the low-frequency region. The frequency dependences of the dielectric loss tangent were characterized by a minimum between two maxima associated with relaxation processes. The frequency dependences of the conductivity of composites were predominantly linear. With an increase in the volume fraction of the filler in the composition of the polymer matrix, the dielectric constant of the composites increased, while the optimal value of the dielectric loss tangent was observed for the composite with filler loading 15 vol.%. It was found that a 1 wt.% addition of silver is sufficient for a noticeable increase in the dielectric constant up to 19 and an acceptable increase in the dielectric loss tangent up to 0.05 at a frequency of 1 kHz.

## Data Availability

Data are contained within the article.
